# Supplementary material for: pH- and concentration-dependent supramolecular assembly of a fungal defensin plectasin variant into helical non-amyloid fibrils
Source: Nat Commun. 2022 Jun 7;13:3162. doi: 10.1038/s41467-022-30462-w (PMC9174238; doi:10.1038/s41467-022-30462-w)
Supplement: Supplementary file 1 — Supplementary Information [file 41467_2022_30462_MOESM1_ESM.pdf]

SUPPLEMENTARY INFORMATION

pH- and concentration-dependent supramolecular assembly of a fungal defensin plectasin variant into helical non-amyloid fibrils

Christin Pohl, Gregory Effantin, Eaazhisai Kandiah, Sebastian Meier, Guanghong Zeng, Werner Streicher, Dorotea Raventos Segura, Per H. Mygind, Dorthe Sandvang, Line Anker Nielsen, Günther H.J. Peters, Christoph Mueller-Dieckmann, Guy Schoen, Allan Noergaard and Pernille Harris

Table S1: Interfacial buried surface area due to fibril formation for all monomers in the asymmetric unit and interface between the protofilaments.

Interaction site	Area (Å^2)	# Buried Atoms	# Surface Atoms	Buried Area Fibril (%)	Buried Area Monomer (%)
au3_1	2423.53	177	127	58.2	29.9
au3_2	2411.54	171	133	56.3	29.3
au3_3	2500.31	175	129	57.6	28.6
au3_4	2427.76	168	136	55.3	28.0
au3_5	2534.86	173	131	56.9	28.3
au3_6	2437.02	169	135	55.6	28.9
au3_7	2495.55	172	132	56.6	28.3
protofilaments	721.67	-	-	-	-

Table S2: Thioflavin T fluorescence end-point measurement on plectasin wildtype, PPI42 and insulin (c=2 mg/mL (450 µM)) in different buffer conditions (excitation: 440±10 nm; emission: 490±10 nm).

sample	buffer	preparation	fluorescence (490 nm)
Insulin	Gly-HCl pH 2.5	dissolved	13428.8
wildtype<br>PPI42	Acetate pH 3.5	dialyzed	-36.4<br>-37.4
wildtype<br>PPI42	Acetate pH 5.5	dialyzed	6.4<br>-12.2
wildtype<br>PPI42	Acetate pH 5.5	diluted	-15.2<br>-13.8
wildtype<br>PPI42	Phosphate pH 6.5	diluted	57.8<br>-1.8

Table S3: Ionic strength of buffers used in this study.

	ionic strength (mM)			
pH	Acetate	Phosphate	Histidine	Citrate
3.5	0.52			7.92
4.0	1.49			11.68
4.5	3.56			16.83
5.0	6.36	10.26	9.13	23.49
5.5	8.47	10.82	7.61	30.05
6.0	9.46	12.4	5	37.32
6.5		16.03	2.4	
7.0		21.54	0.91	
7.5		26.24	0.3	
8.0		28.64	0.24	

Table S4: Summary of cryo-EM data collection and atomic model statistics of PPI42.

**Data collection**		
Microscope	Krios G3 (ThermoFischer Scientific)	
Voltage (kV)	300	
Magnification<br>Unbinned pixel size	165,000x<br>0.827 Å/pixel	
Camera	K2 Summit (Gatan Inc)	
Exposure time	4s	
Number of frames	40	
Total dose ($e^{-}/Å^{2}$)	46.4	
**Image processing**		
	Mature Fibril	Isolated Protofilament
EMDB	EMD-12775	EMD-12776
Rotational symmetry	C2	C3
Helical symmetry (axial rise/azimuthal angle)	25.1 Å / 16.75°	3.76 Å / 156.5°
Final number of Particles	764822	66272
Map resolution in Å (FSC 0.143)	1.97	3.35
**Model statistics**		
PDB	7OAE	7OAG
Model resolution in Å (FSC 0.5)	2.15	3.5
Ramachandran favored (%)	97.4	94.7
Ramachandran outliers (%)	0.0	0
Rama Z score	0.22	-0.35
Rotamer outliers (%)	0.0	0
C-beta deviations	0	0
Rms on bond lengths	0.0076	0.0069
Rms on bond angles	0.53	0.67
Clashscore	0.99	5.52
Molprobity score	0.91	1.66

Table S5: Summary of X-ray data collection and refinement of wildtype plectasin.

**Data collection**	
Space group	$P2_1$
Unit cell	
a, b, c (Å)	23.68, 20.09, 32.55
α, β, γ (°)	α= 90, β=104.28, γ=90
Total number of reflections	59453(3272)
Number of unique reflections	10216(1020)
Protein molecules in ASU	1
Resolution limits (Å)	31.53-1.13(1.20-1.13)
R_{merge}	0.033(0.343)
R_{pim}	0.018(0.274)
CC(½)	0.999(0.900)
Completeness (%)	89.9(54-6)
Average I/σ (I)	22.49 (2.65)
Wilson plot B-factor (Å^2)	17.38
Multiplicity	6.0(3.6)
**Refinement**	
R_{work}	0.1459
R_{free}	0.1762
Number of reflections	9708
Reflections used for R-free	507
Number of non-hydrogen atoms in ASU	320
Number of water molecules	25
No non-hydrogen atoms in ligand	7
**Root mean square deviation from ideal**	
Bond lengths (Å)	0.018
Bond angles (°)	2.005
B-factors (Å^2)	26.43
Solvent content	
MolProbity clash score, all atoms*	16
Overall MolProbity score	2.4

* defined as the number of steric overlaps > 0.4Å per thousand atoms. MolProbity score is poor due to clashes of the protein with PEG molecule present in the crystal, indicating that this molecule is more flexible and not so well defined as the protein. No protein-protein clashes are observed.

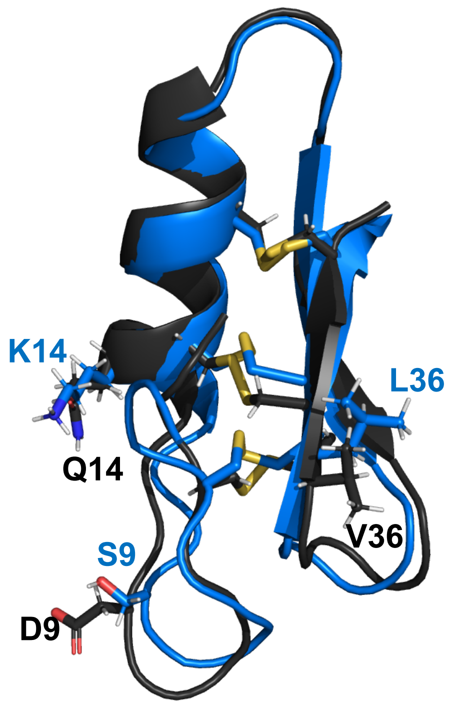

		1 10 20 30 40
**wildtype**	-	GFGCNGPWDEDDMQCHNHCKSIKGYKGGYCAKGGFVCKCY
**PPI42**	-	GFGCNGPW**S**EDDM**K**CHNHCKSIKGYKGGYCAKGGF**L**CKCY

Figure S1: Structure of plectasin wildtype (black) (PBDID: 3E7U[1]) and energy-minimized structure of PPI42 (blue)[2] with mutated amino acids and three disulfide bonds (C4-C30; C15-C37; C19-C39) shown as sticks. The sequence is shown with mutated amino acids labeled in red for PPI42. Figure was made with PyMOL[3].

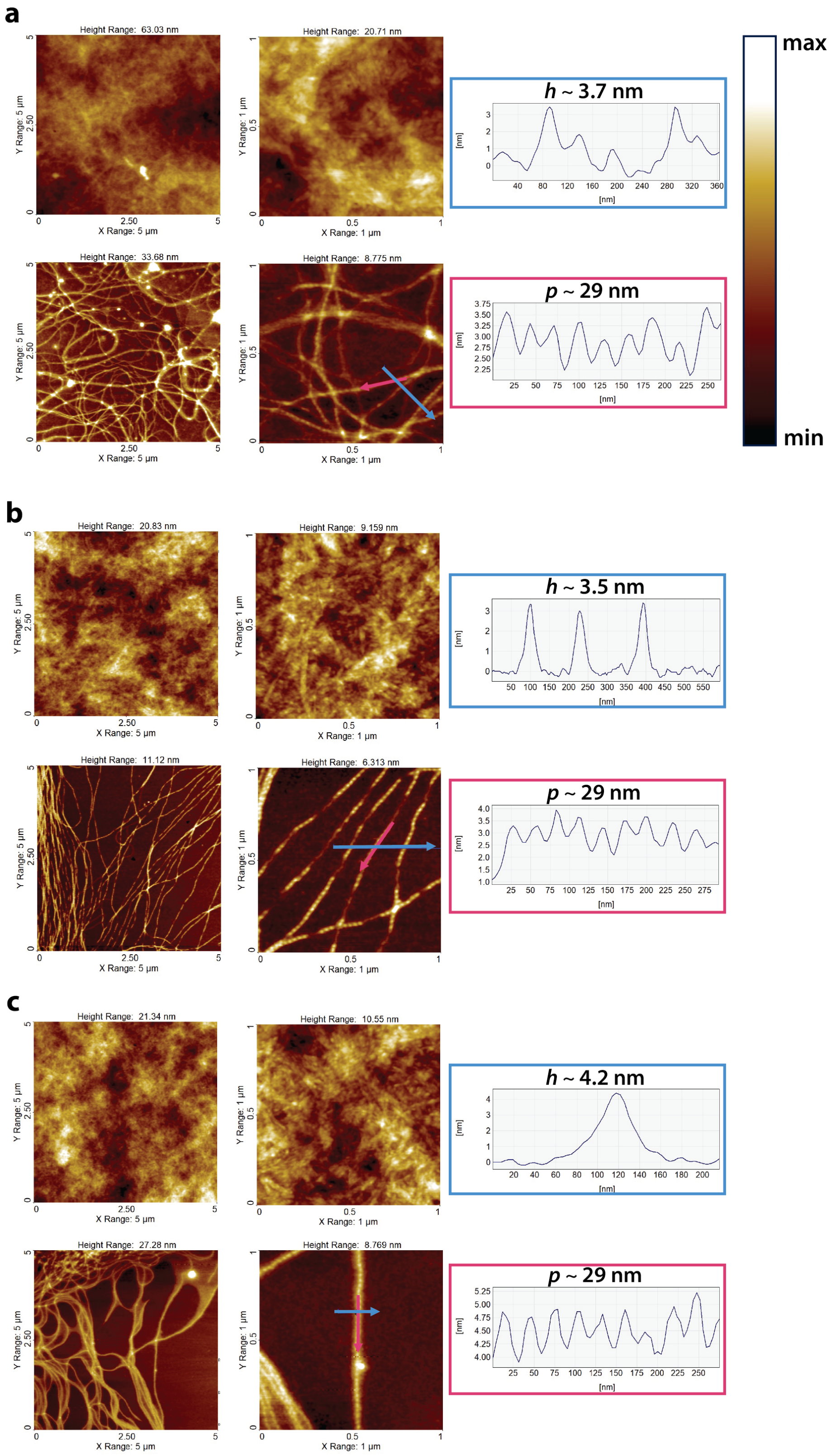

Figure S2: a: AFM measurements on PPI42 fibrils in histidine buffer pH 6.5 (n=2, representative images shown). b: AFM measurements on PPI42 fibrils in citrate buffer pH 5. c: AFM measurements of PPI42 fibrils in H_2O pH ≈ 5. The respective measured height (*h*, blue box measured along the blue arrow) and the periodicity along the fibril (*p*, red box measured along the red arrow) are shown on the right. Measured range is shown on the figure axis. Protein concentration for all measurements was approx. 20 mg/mL (4.5 mM).

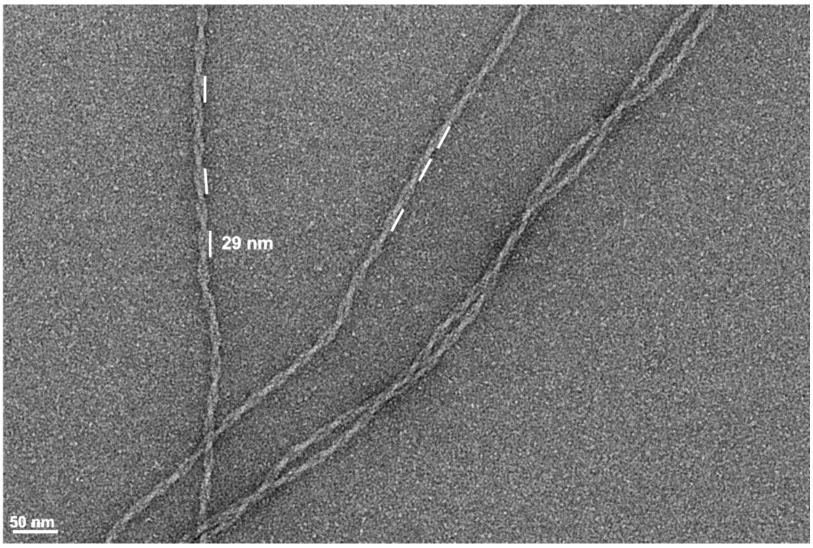

Figure S3: Representative measurement of periodicity along the fibril on images obtained with negative stain EM. Measurement was performed in acetate buffer pH 5.5 with a protein concentration of approx. 20 mg/mL (4.5 mM).

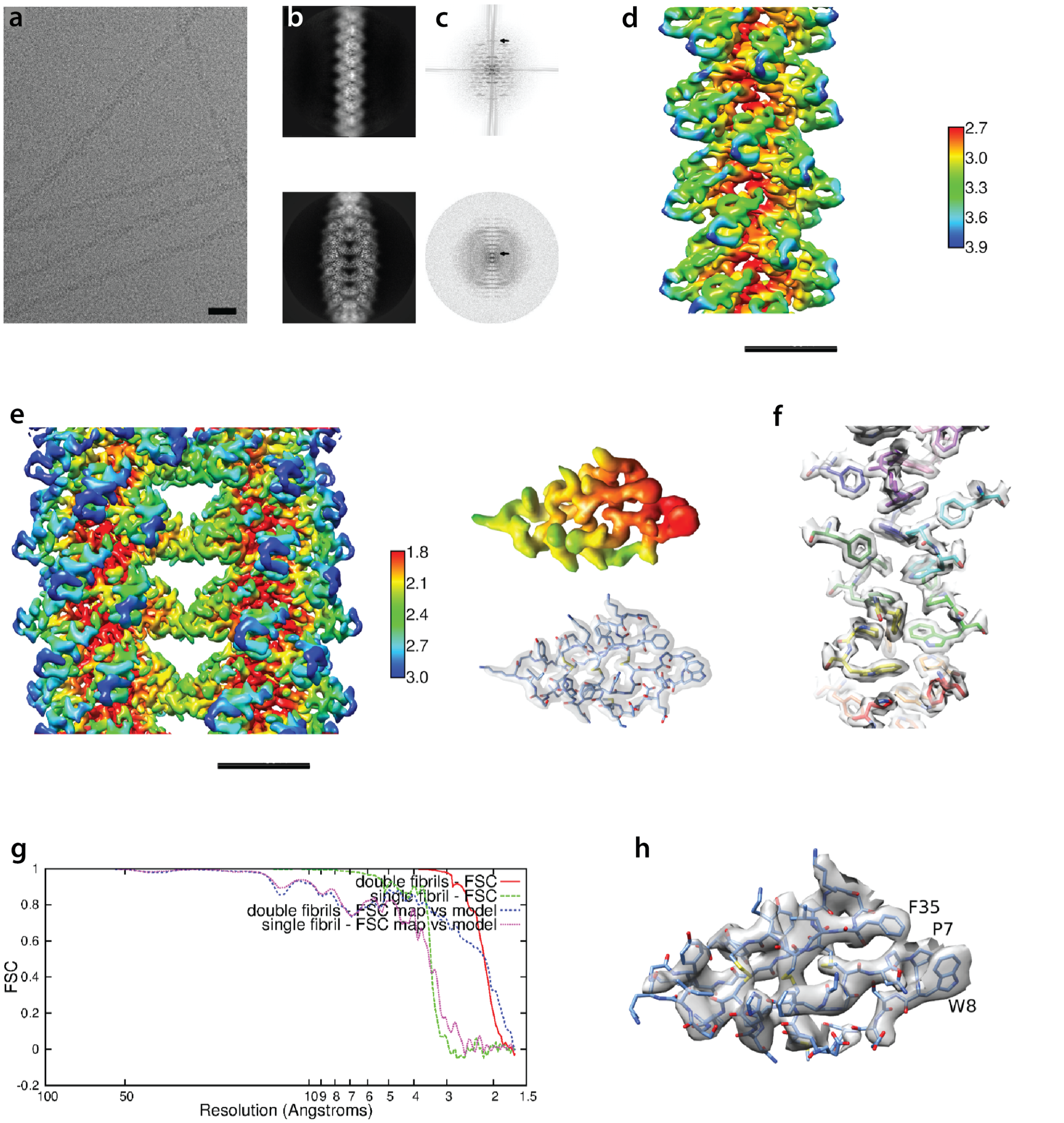

Figure S4: a: Cryo-EM field of view of PPI42 fibrils (n>3, representative images shown). Scale bar represents 200 Å. b: Exemplary 2D class averages. Upper panel: isolated protofilaments; lower panel: mature fibril. c: Power spectra of the two class averages shown in b. Arrows point to layer lines at 24.8 Å and 3.8 Å for the isolated protofilaments and mature fibril, respectively. d,e: Local resolution maps for the isolated protofilament (d) and mature fibril (e) reconstructions. Scale bar represents 30 Å. e: Local resolution map for one plectasin monomer in the mature fibril and it's corresponding atomic model. Color code indicates resolution in Å. f: Illustration of the high-resolution features of the mature fibril reconstruction (shown in e). The hydrophobic core (P7, W8 and F35) of a single protofilament of the mature fibril is displayed. PPI42 monomers are colored individually. Cryo-EM Coulomb potential map is shown in gray. g: Fourier Shell Correlation (FSC) curves: gold standard FSCs between two independent 3D reconstructions of the single fibril (green dotted line) and fibril superstructure (red line) and FSC between the cryo-EM maps and the refined atomic models for both the single fibril (dotted pink line) and fibril superstructure (dotted blue line). h: Atomic model of PPI42 monomer built in the isolated protofilament reconstruction (3.35 Å overall resolution). Hydrophobic core residues P7, W8 and F35 are indicated.

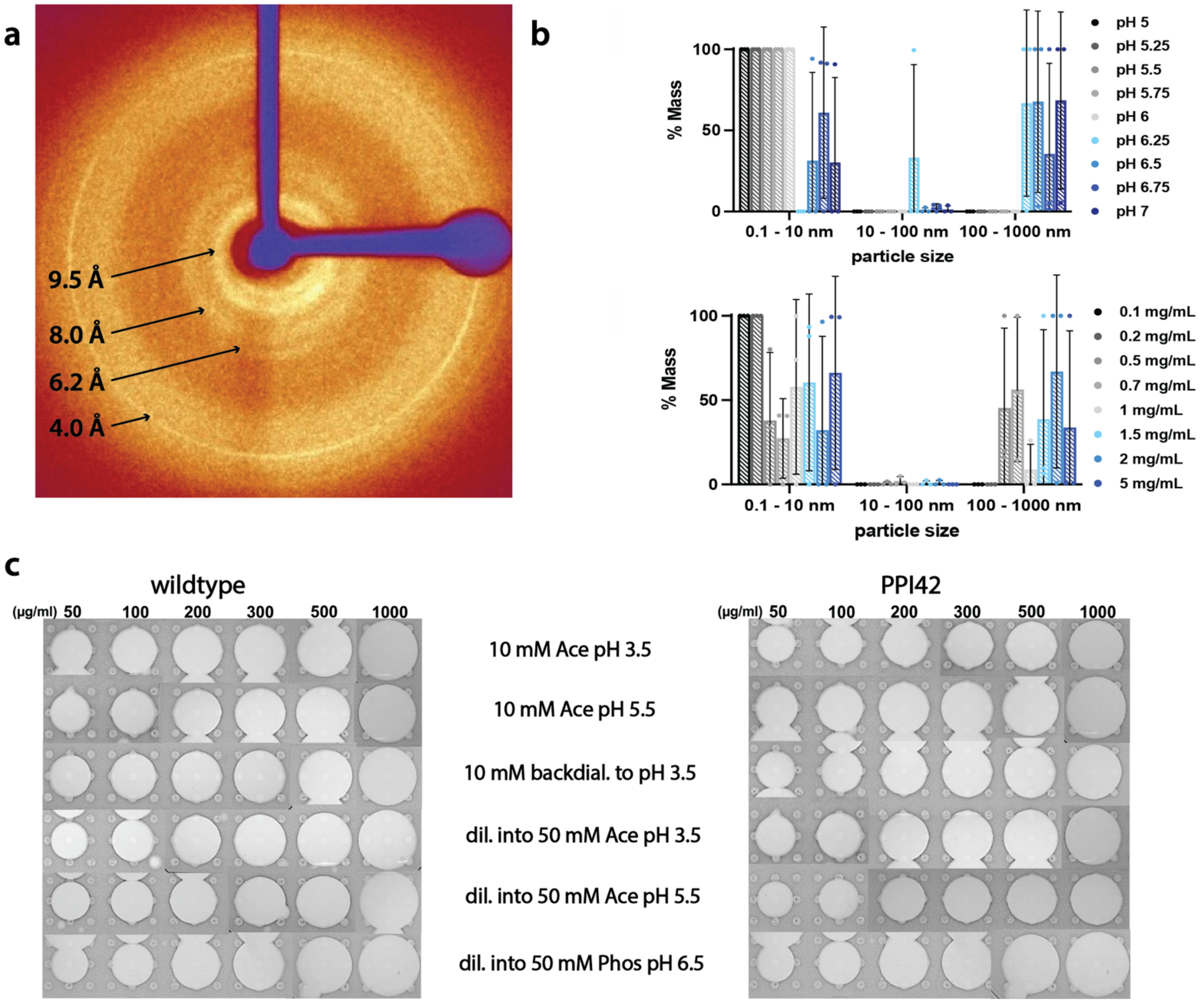

Figure S5: Characterization of fibrils formed by PPI42. a: Fiber diffraction image of the plectasin fibrils of PPI42. The relevant diffraction signals and distances are highlighted. Measurement was performed on dried fibrils in acetate buffer pH 5.5 with a protein concentration of approx. 20 mg/mL (4.5 mM). b: Dependence of PPI42 oligomerization on pH (upper panel) and protein concentration (lower panel) determined by DLS. pH dependency was performed in phosphate buffer (protein concentration 2 mg/mL (450 µM)). Concentration dependency was performed in phosphate buffer pH 6.5. Data is shown as %Mass. Bars represent mean ± S.D. for 3 replicates (shown as dots). Source data are provided as a Source Data file. c: Anti-microbial activity of the plectasin wildtype and PPI42 measured by semi-quantitative radial diffusion assay. Clearing zones to measure the anti-microbial activity of the plectasin wildtype and PPI42 at different protein concentration in different conditions.

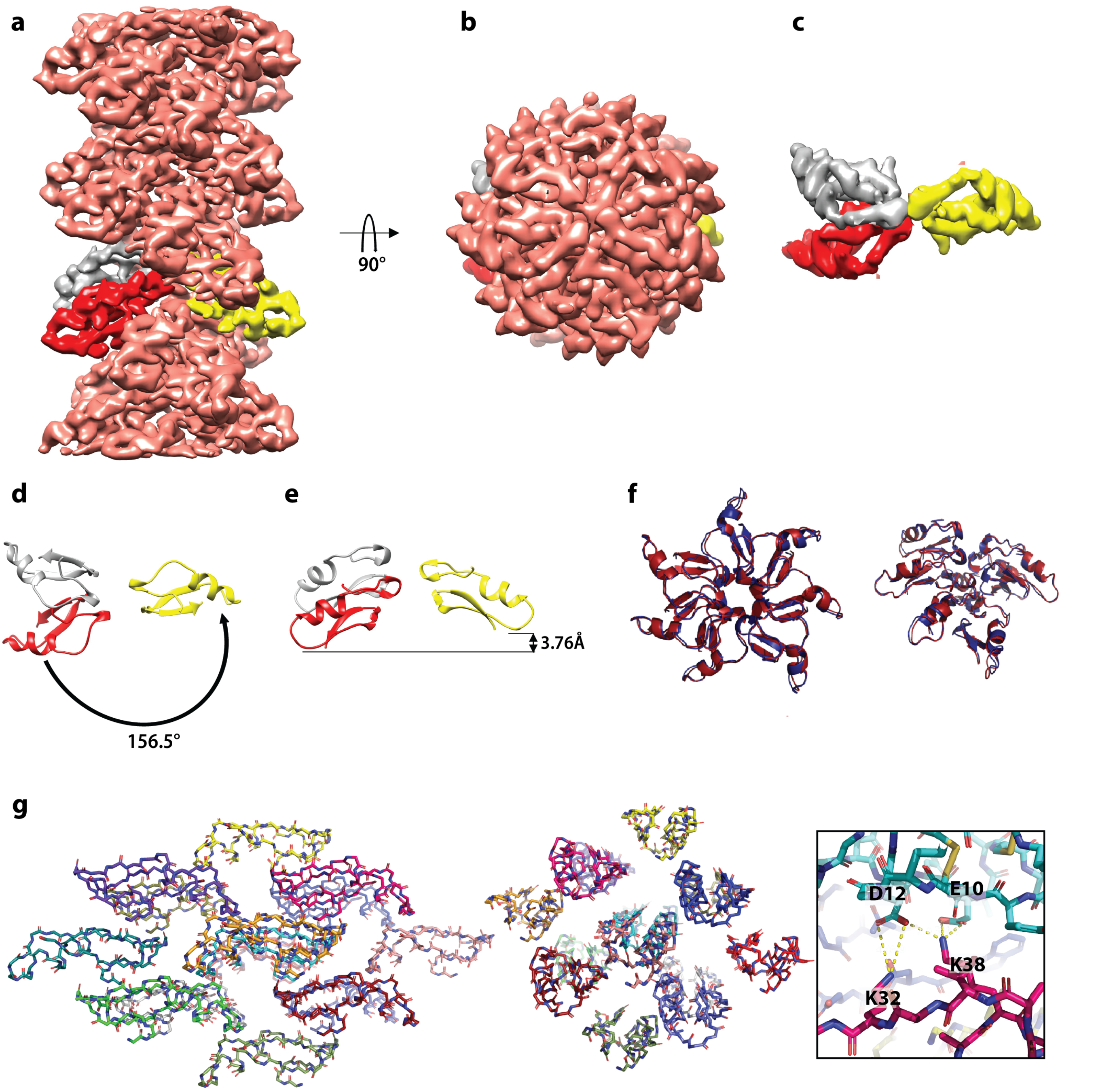

Figure S6: a-c: Cryo-EM map of the isolated protofilament formed by PPI42 at a resolution of 3.35 Å. Isosurface representation of the cryo-EM map from the side (a) and top (b). c: Three consecutive monomers related by helical symmetry (red to yellow to gray), shown in top view. d,e: Atomic model views from the top (d) and from the side (e) of three consecutive monomers of the PPI42 protofilament. The structure consists of a right-handed helix with an axial rise of 3.76 Å and an azimuthal angle of 156.5°. f: Superposition of of the atomic model of the mature fibril and isolated protofilament. Seven monomers that form the asymmetric unit in the mature fibril are shown. Monomers of the isolated protofilament are shown in red and of the mature fibril in blue. The all-atom RSMD is 0.727 Å. g: Crystal arrangement of the plectasin wildtype at 1.1 Å resolution from the front (left) and from the top (middle). Conserved crystal contacts of the acidic amino acid stretch D9, E10, D11, D12 with lysines are shown in detail (right).

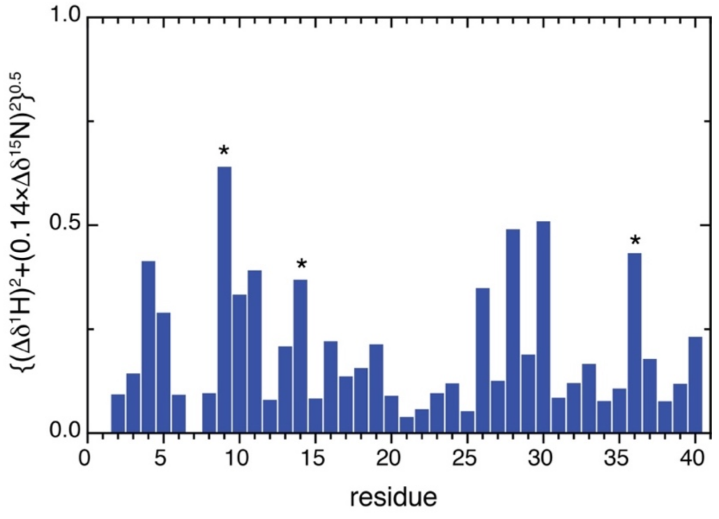

Figure S7: Chemical shift differences for ^{1}H-^{15}N groups of plectasin wildtype and PPI42 at 298 K (acetate buffer pH 4.5 at 1.1 mg/mL (250 μM) protein concentration). Changes in ^{15}N chemical shifts were weighted according to the bigger ^{15}N (x0.14) than ^{1}H chemical shift range. The three mutated amino acids are labeled with asterisks.

Supplementary Discussion

To investigate structural changes between PPI42 and plectasin wildtype, we compared their chemical shift differences. Despite the lack of isotope-enriched protein, we were able to conduct a full assignment of backbone amide groups (^{1}H-^{15}N groups) at natural isotopic enrichment using cryogenically cooled detection electronics and high-field NMR instrumentation. The three mutated residues are marked by asterisks in Figure S7 and unsurprisingly these residues show some of the largest changes in chemical shifts between wildtype and PPI42. We observed significant conformational changes between the plectasin wildtype and PPI42 in E10 and D11 (Figure S7), which are involved in the fibril stabilisation and positioned in the core of the protofilament. Due to the D9S mutation, N5 shows different coordination in PPI42 and wildtype and accordingly a significant difference in chemical shift. Adjacent to the monomer interface of M13/H16 with F2/H18 are further K26 and G28, which accordingly also show significant changes in local structure and/or dynamics between PPI42 and wildtype. The last residues with strong changes in backbone chemical shifts are C4 and C30, indicating that disulfide bond conformations may differ between the two constructs. We concur that the mechanistic role of these residues in nucleation could be further scrutinized through heteronuclear relaxation and relaxation dispersion experiments in NMR studies dedicated to the dynamic changes to protein chain upon nucleation and fibrillation.

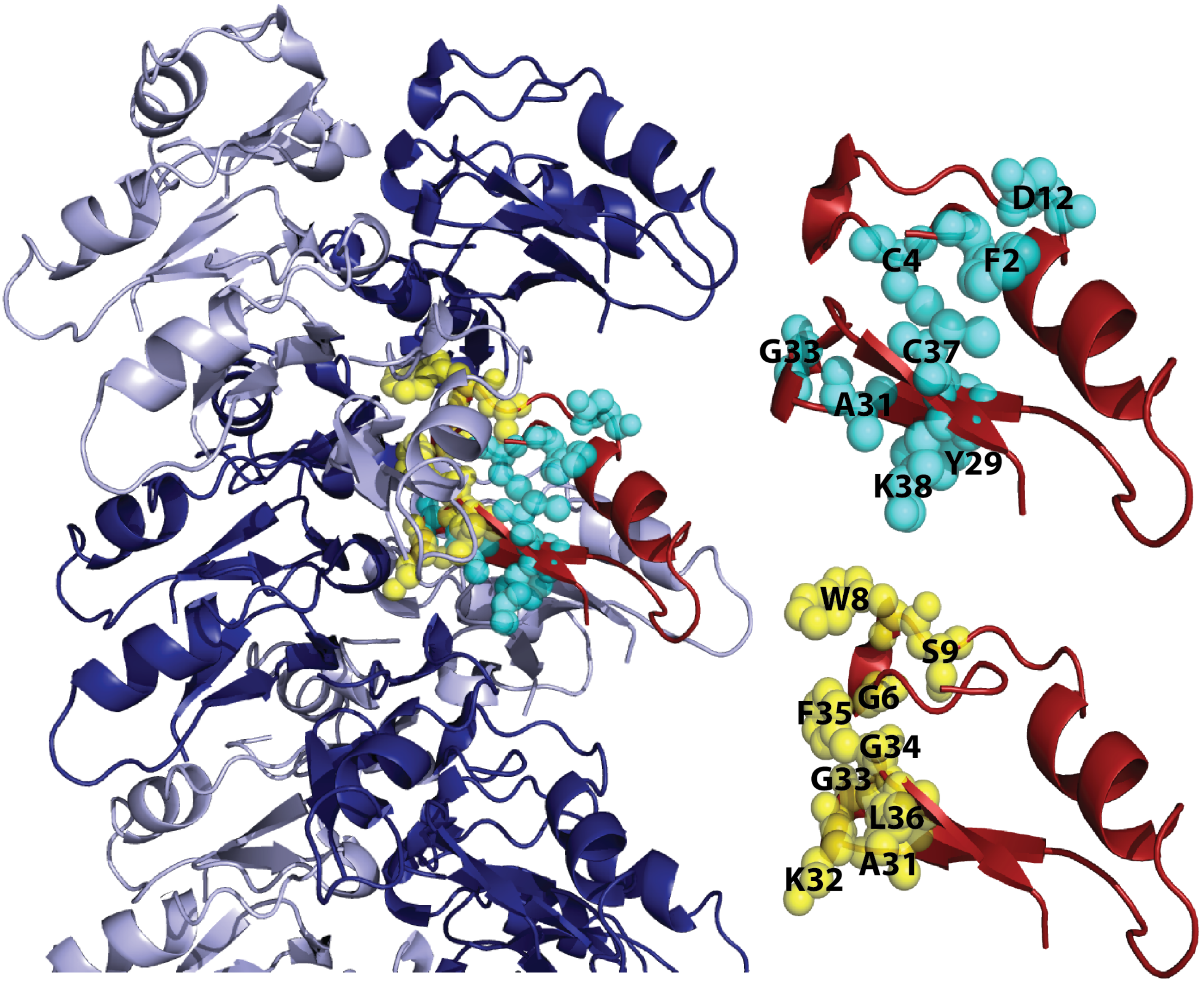

Figure S8: Proposed binding site[4] of plectasin to membrane (yellow) and Lipid II (cyan) within the fibril (one protofilament of the mature fibril).

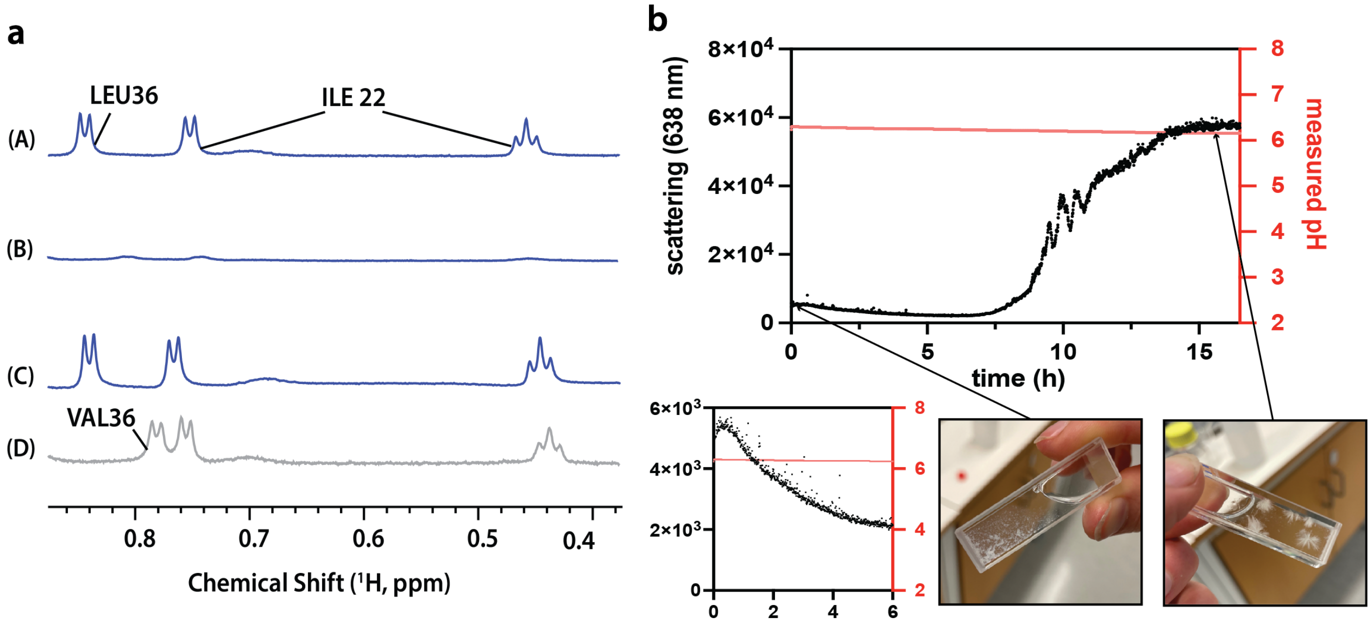

Figure S9: a: ¹H NMR spectra for PPI42 at 1.1 mg/mL (250 μM) protein concentration in acetate buffer pH 3.5 (A), at equilibrium in phosphate buffer at pH 7.0 (B) and after titration with 1 M HCl from pH 7.0 to pH 3.0 (C). The spectra show that fibrillation is reversible. Signal intensities for the non-fibril forming wildtype at comparable concentrations is shown in (D). b: Light scattering and monitored pH of plectasin wildtype diluted into phosphate buffer pH 7 at a protein concentration of 1.1 mg/mL (250 μM). Insert shows the decrease in light scattering after dilution due to sedimentation of aggregates with time. Pictures show the sample directly after adding protein stock solution to buffer pH 7 (visible aggregation) and after the measurement where crystals have formed. Source data are provided as a Source Data file.

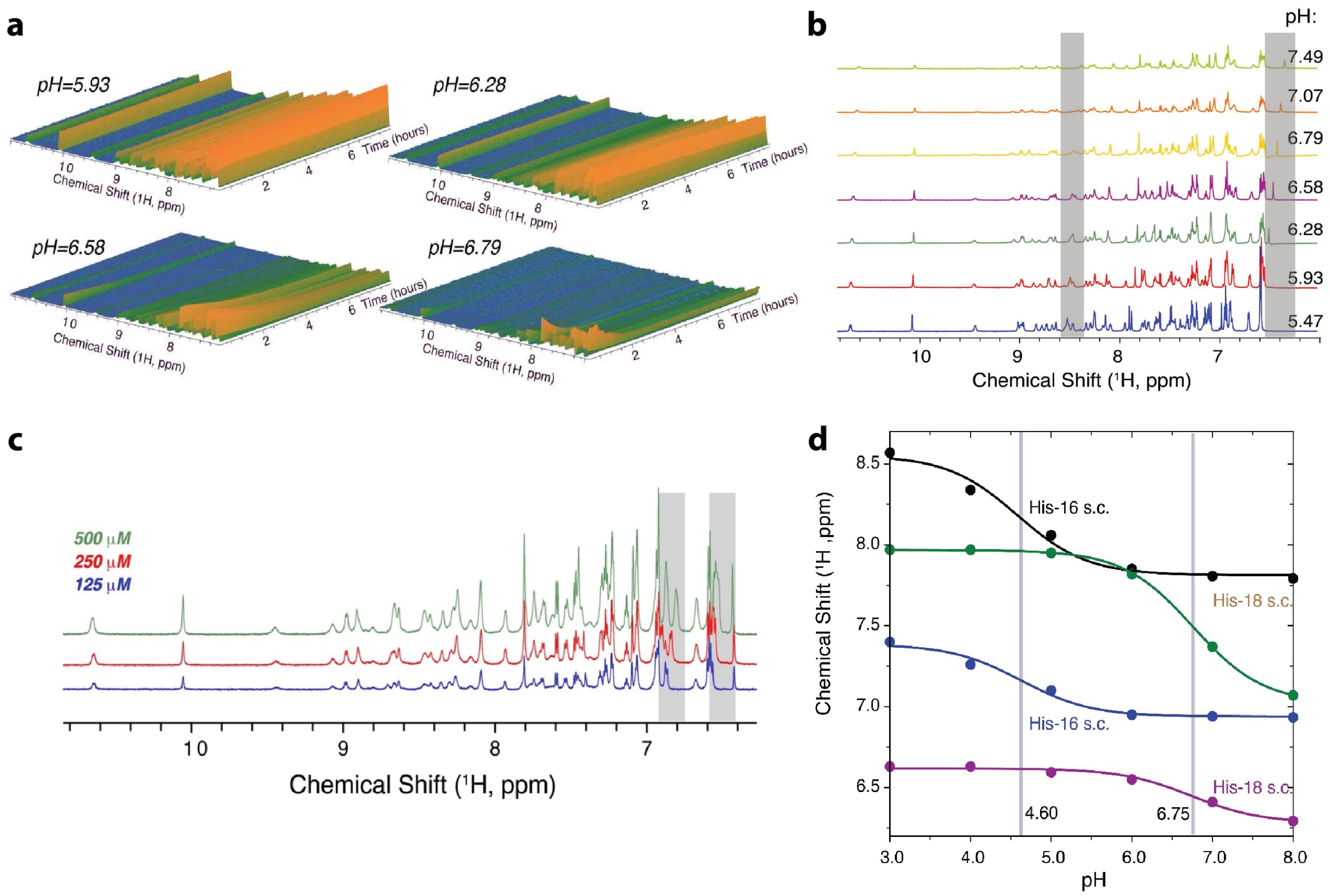

Figure S10: a: Loss of monomer signals in NMR over time for PPI42 (c=0.25 µM, 298 K) at different pH (phosphate buffer). b: ^{1}H chemical shift of PPI42 in a series of pH from pH 5.5 to 7.5. c: ^{1}H NMR spectra of PPI42 at pH 6.75 at different protein concentrations. The area, where a change in NMR signals is most evident is shaded in grey. d: Titration curves of wildtype plectasin H16 and H18 (H^2 and H^4 in imidazole ring) determined by resolving the signals with 2D ^{1}H-^{1}H TOCSY. The grey bars indicate the pK_a of the titratable side chains. All measurements were conducted in phosphate buffer.

REFERENCES

1. Mandal, K. *et al.* Racemic crystallography of synthetic protein enantiomers used to determine the X-ray structure of plectasin by direct methods. *Protein Sci.* **18**, 1146–1154 (2009).

2. Pohl, C. *et al.* The effect of point mutations on the biophysical properties of an anti-microbial peptide: development of a screening protocol for peptide stability screening. *Mol. Pharm.* **17**, 3298–3313 (2020).

3. Schrödinger, LLC. *The {PyMOL} Molecular Graphics System, Version~1.8*. (2015).

4. Schneider, T. *et al.* Plectasin, a fungal defensin, targets the bacterial cell wall precursor lipid II. *Science (80-.).* **328**, 1168–1172 (2010).
