# Supplementary material for: pH- and concentration-dependent supramolecular assembly of a fungal defensin plectasin variant into helical non-amyloid fibrils
Source: Nat Commun. 2022 Jun 7;13:3162. doi: 10.1038/s41467-022-30462-w (PMC9174238; doi:10.1038/s41467-022-30462-w)
Supplement: Supplementary file 3 — Reporting Summary [file 41467_2022_30462_MOESM3_ESM.pdf]

nature research

Corresponding author(s): Christin Pohl; Pernille Harris

Last updated by author(s): Feb 28, 2022

Reporting Summary

Nature Research wishes to improve the reproducibility of the work that we publish. This form provides structure for consistency and transparency in reporting. For further information on Nature Research policies, see our Editorial Policies and the Editorial Policy Checklist.

Statistics

For all statistical analyses, confirm that the following items are present in the figure legend, table legend, main text, or Methods section.

n/a	Confirmed	
☐	☒	The exact sample size (*n*) for each experimental group/condition, given as a discrete number and unit of measurement
☐	☒	A statement on whether measurements were taken from distinct samples or whether the same sample was measured repeatedly
☒	☐	The statistical test(s) used AND whether they are one- or two-sided<br>*Only common tests should be described solely by name; describe more complex techniques in the Methods section.*
☒	☐	A description of all covariates tested
☒	☐	A description of any assumptions or corrections, such as tests of normality and adjustment for multiple comparisons
☐	☒	A full description of the statistical parameters including central tendency (e.g. means) or other basic estimates (e.g. regression coefficient) AND variation (e.g. standard deviation) or associated estimates of uncertainty (e.g. confidence intervals)
☒	☐	For null hypothesis testing, the test statistic (e.g. *F*, *t*, *r*) with confidence intervals, effect sizes, degrees of freedom and *P* value noted<br>*Give P values as exact values whenever suitable.*
☒	☐	For Bayesian analysis, information on the choice of priors and Markov chain Monte Carlo settings
☒	☐	For hierarchical and complex designs, identification of the appropriate level for tests and full reporting of outcomes
☒	☐	Estimates of effect sizes (e.g. Cohen's *d*, Pearson's *r*), indicating how they were calculated

Our web collection on statistics for biologists contains articles on many of the points above.

Software and code

Policy information about availability of computer code

Data collection	Topspin 3.5 pl7 was used for NMR data collection. Spectra Manager CFR 2.10.01 was used for CD data collection on a Jasco J-1000 spectrometer. FLUOstar Omega v. 5.11 was used to monitor ThT fluorescence carried out on a FLUOstar omega multi-mode microplate reader. DLS was measured with a DynoPro Plate reader using DYNAMICS 7.8.1.3 for instrument control and analysis. Negative staining was performed using Tecnai 12 LaB6 electron microscope at 120 kV accelerating voltage using CCD Camera Gatan Orius 1000. Fiber diffraction was performed using a Supernova CCD diffractometer. cryo-EM measurements were performed on a Titon Krios G3 using automatic image recording on a K2 summit direct detector in counting mode with EPU. AFM was measured using a Multimode 8 AFM with PeakForce Quantitative Nanomechanical Mapping with TAP150A probes.
Data analysis	cryo-EM: RELION 3.1, MotionCor2 1.3.2, GCTF 1.18, CHIMERA 1.15, CHIMERAX 1.2.5, ROSETTA2, PHENIX 1.18.2, COOT 0.8.9, Pymol 2.4.2 ; DLS: DYNAMICS 7.8.1.3; NMR: proFit 7; X-ray Crystallography: autoPROC toolbox 1.0.5, XDS/XSCALEv.Jan 31, 2020, Pointless,1.11.21, CCP4 program suite7.1.010, Molrep11.5.05, REFMAC5.8.0267; Figures and Images: Origin® 2019, GraphPad Prism 9.3.1, ImageJ 1.8.0

For manuscripts utilizing custom algorithms or software that are central to the research but not yet described in published literature, software must be made available to editors and reviewers. We strongly encourage code deposition in a community repository (e.g. GitHub). See the Nature Research guidelines for submitting code & software for further information.

Data

Policy information about availability of data

All manuscripts must include a data availability statement. This statement should provide the following information, where applicable:

- Accession codes, unique identifiers, or web links for publicly available datasets
- A list of figures that have associated raw data
- A description of any restrictions on data availability

The authors declare that the data supporting the findings of this study are available within this publication. Source data are provided with this paper for figures 5a-f, 6a,b, S5b and S9b. Additional raw data is available from the corresponding author upon reasonable request. The atomic structures determined in this study are deposited in the PDB database and the EM data bank. PDB identifier for mature fibril is 7OAE [https://doi.org/10.2210/pdb7OAE/pdb], for the isolated protofilament 7OAG [https://doi.org/10.2210/pdb7OAG/pdb], for the plectasin wildtype crystal structure 7O76 [https://doi.org/10.2210/pdb7O76/pdb]. EMBD identifier for mature fibril is EMD-12775, for the isolated protofilament EMBD-12776.

Field-specific reporting

Please select the one below that is the best fit for your research. If you are not sure, read the appropriate sections before making your selection.

- [x] Life sciences
- [ ] Behavioural & social sciences
- [ ] Ecological, evolutionary & environmental sciences

For a reference copy of the document with all sections, see nature.com/documents/nr-reporting-summary-flat.pdf

Life sciences study design

All studies must disclose on these points even when the disclosure is negative.

Sample size	Sample size for cryo-EM and X-ray Crystallography are indicated in supplementary table 4 and 5. For X-ray crystallography sample size was used based on the size of the crystal, measured under a microscope. The crystal diffraction was 1.1-1.2 Angstrom, making this sample size optimal. For cryo-EM sample 8443 micrographs were automatically recorded based on the measurement time constrains. 7241 micrographs were used for structure determination, which is considered sufficient and resulted in a high enough resolution for unambiguous structure determination of the high-resolution structure. Sample size for other methods are described in method section and are generally considered sufficient for all methods used in this study.
Data exclusions	Cryo EM images were excluded based on CTF estimation step excluding micrographs with an estimated resolution lower than 4.5 A (pre-established). An initial set of articles were manually picked followed by automatic picking using a conservative threshold. DLS autocorrelation curves were excluded upon manual inspection using pre-established exclusion criteria.
Replication	AFM was performed on 4 independent samples in different buffer conditions in 2 independent samples. CD spectra were recorded on 3 independent replicas and accumulated over 5 recorded spectra. Fibril formation for cryo-EM was ensured in 3 independent samples. DLS was measured in triplicates in 3 independent samples. Fiber Diffraction was performed in two independent samples. Negative staining was performed in two independent samples. NMR measurements were recorded on 3 independent samples as a series of 1D spectra performed on a series of samples in different pH. Gel elasticity assay was performed on 3 independent samples. Fibril formation reversibility assay was performed on 3 independent samples. All attempts to replicate the results were successful.
Randomization	not applicable because no human or animal participation was used in this study.
Blinding	not applicable because sample conditions needed careful control to achieve fibril formation.

Reporting for specific materials, systems and methods

We require information from authors about some types of materials, experimental systems and methods used in many studies. Here, indicate whether each material, system or method listed is relevant to your study. If you are not sure if a list item applies to your research, read the appropriate section before selecting a response.

Materials & experimental systems

n/a	Involved in the study	
☒	☐	Antibodies
☒	☐	Eukaryotic cell lines
☒	☐	Palaeontology and archaeology
☒	☐	Animals and other organisms
☒	☐	Human research participants
☒	☐	Clinical data
☒	☐	Dual use research of concern

Methods

n/a	Involved in the study	
☒	☐	ChIP-seq
☒	☐	Flow cytometry
☒	☐	MRI-based neuroimaging
